# Expression and Regulatory Roles of SKAP2 and Cortactin in Mouse Ovarian Tissue and Oocyte Maturation

**DOI:** 10.1007/s43032-025-01925-4

**Published:** 2025-07-08

**Authors:** Ying Feng, Xinyi Hang, Zhumei Sheng, Yu Chen, Xuejing Jin

**Affiliations:** 1 Department of Fourth Clinical Medical College, Zhejiang Chinese Medical University, Hangzhou, 310053 Zhejiang China; 2 Department of Women’s Health Division, Hangzhou Women’s Hospital, Hangzhou, 310008 Zhejiang China; 3 Department of Affiliated Hospitals (School of Clinical Medicine, School of Stomatology), Hangzhou Normal University, Hangzhou, 310022 Zhejiang China; 4 Department of the Reproductive Endocrinology Division, Hangzhou Women’s Hospital, Hangzhou, 310008 Zhejiang China

**Keywords:** Oocyte maturation, Src kinase-associated phosphoprotein 2, Cortactin, Meiosis

## Abstract

Oocyte maturation is a critical phase in mammalian reproduction. Src kinase-associated phosphoprotein 2 (SKAP2) and cortactin, both cytoskeletal regulatory proteins that play pivotal roles in cell motility and signal transduction. However, their specific functions and mechanisms during oocyte maturation are currently unknown. This study revealed the roles of SKAP2 and cortactin in the oocyte maturation process. To investigate the distribution, activity, and functional significance of SKAP2 during murine oocyte asymmetric division, ovarian tissues were infected with lentiviruses expressing SKAP2 and cortactin shRNA. Cell morphology was analyzed to assess the expression and distribution of cyclins, SKAP2, Wiskott–Aldrich syndrome protein family member 2 (WAVE2), and cortactin. Additionally, the levels of SKAP2, Cyclin A, Cyclin B, Cyclin-dependent kinase 1 (CDK1), and Polo-like kinase 1 (PLK1) were quantified using qPCR and western blotting. SKAP2 was highly expressed in ovarian tissues and oocytes. Knockdown of SKAP2 significantly impaired oocyte maturation and altered the expression of key cell cycle proteins. SKAP2 regulates oocyte maturation and division by modulating cell cycle proteins and interacting with WAVE2 and cortactin. SKAP2 and cortactin are important in the regulatory mechanisms of oocyte maturation, these findings offer potential targets and strategies for advancing reproductive technologies and treating reproductive disorders.

**Supplementary Information:**

The online version contains supplementary material available at 10.1007/s43032-025-01925-4.

## Introduction

The oocyte, one of the largest cells in mammals, serves as the female gamete essential for reproduction. Oocyte maturation encompasses two key processes: maturation of nuclear and cytoplasmic. Nuclear maturation involves chromosomal segregation, reflecting the oocyte's capacity to resume meiosis, and occurs during the follicular development stage [1]. In contrast, cytoplasmic maturation provides the mRNA, proteins, and substrates necessary for subsequent fertilization and early embryonic development [1, 2]. During meiosis, the oocyte undergoes germinal vesicle breakdown (GVBD), asymmetric cytoplasmic division, and the release of the first polar body, ultimately forming a haploid metaphase II (MII) oocyte capable of fertilization [3].

Src kinase-associated phosphoprotein 2 (SKAP2), also known as SKAP55 homolog (SKAP55-HOM), is a substrate of Src family kinases and functions as an intracellular adaptor protein [4–6]. Structurally, SKAP2 comprises a pleckstrin homology (PH) domain, a C-terminal Src homology 3 (SH3) domain, multiple tyrosine phosphorylation sites, and an N-terminal coiled-coil structural domain [7]. Previous studies have showed that SKAP2 interacts with and modulates the activity of various proteins in oocytes, facilitating the assembly of signaling complexes and regulating diverse cellular functions [6, 8].

Cortactin, encoded by the *CTTN* gene located at chromosome band 11q13, is an actin-binding protein whose primary function is to regulate cytoskeletal rearrangements in eukaryotes [9–11]. It consists of four functional domains: an N-terminal acidic (NTA) domain, an F-actin-binding domain, a proline-rich region, and a C-terminal SH3 domain [11, 12]. Cortactin orchestrates cytoskeletal dynamics by interacting with multiple cytoskeletal and signaling proteins through its SH3 domain. Additionally, cortactin is a target of Src family tyrosine kinases, and phosphorylation of residues within its proline-rich region by SKAP2 enhances intracellular signaling [13, 14].

Previous studies have shown that SKAP2 interacts with WAVE2 in mouse oocytes to regulate ARP2/3 activity, a critical process for actin-dependent asymmetric cytoplasmic division [15]. WAVE2, officially designated as WASF2 within the Wiskott-Aldrich syndrome protein family, functions as a crucial cytoskeletal component that interacts with actin filaments [16, 17]. As a core component of the WAVE regulatory complex, WAVE2 initiates actin polymerization, making it a key regulator of cytoskeletal dynamics [16, 18, 19].

This study systematically investigated the transcriptional profile, subcellular localization, and biological functions of SKAP2 during meiotic progression. Experimental results demonstrated that SKAP2 forms a molecular complex with WAVE2 to regulate Arp2/3 activity during mouse oocyte maturation, a mechanism essential for establishing cytoplasmic asymmetry and polar body extrusion. These findings highlight the critical role of SKAP2 in oocyte maturation and provide novel insights into the regulatory mechanisms of cytoskeletal dynamics during mammalian reproduction.

## Materials and Methods

### Oocyte Acquisition and Culture

All experimental procedures involving animal subjects were performed following the National Institutes of Health's ethical standards. The study protocol received official authorization from the Institutional Animal Care and Use Committee prior to implementation. Germinal vesicle (GV)-stage oocytes were isolated from the ovarian tissues of 4–6-week-old female mice of the imprinted control region (ICR) strain. The collected oocytes were cultivated in M2 medium (Sigma-Aldrich, Missouri, USA) under controlled conditions at 37 °C with 5% CO_2_. Oocytes at various developmental stages were collected for subsequent experiments [20].

### Protein Blotting

For protein analysis, 200 oocytes from each experimental group were homogenized in sodium dodecyl sulfate (SDS) sample buffer and heat-denatured at 100 °C for 5 min. The proteins were then separated by SDS-PAGE and transferred onto polyvinylidene fluoride (PVDF) membranes. To minimize background interference, the membranes underwent blocking treatment for 1 h under ambient conditions using Tris-buffered saline (TBS) buffer containing 5% skim milk and 0.1% Tween-20 detergent. Immunoblotting was then performed by incubating with primary antibodies at 4 °C for 12–16 h, using the specified antibody concentrations: mouse monoclonal anti-actin (1:1000), rabbit polyclonal anti-SKAP2 (1:1000), rabbit polyclonal anti-WAVE2 (1:1000), and rabbit polyclonal anti-Arp2 (1:500). After three 10-min washes with TBST (TBS with 0.1% Tween-20), the membranes were cultivated with species-specific horseradish peroxidase (HRP)-conjugated secondary antibodies (mouse anti-rabbit IgG and rabbit anti-mouse IgG, both at 1:10,000 dilution) at room temperature for 1 h. Following additional TBST washes, protein signals were detected using enhanced chemiluminescence (ECL) reagents [21]. Clones and conjugation or secondary antibodies of the antibodies used in the study are as follows. Rabbit polyclonal anti-SKAP2 antibody was purchased from Proteintech Group (Chicago, CA, USA). Rabbit polyclonal anti-ARP2 antibody and mouse monoclonal anti-actin antibody were purchased from Abcam (Cambridge, UK). Rabbit polyclonal anti-WAVE2 antibody was purchased from Santa Cruz (Santa Cruz, CA, USA). Horseradish peroxidase (HRP)-conjugated secondary antibodies (mouse anti-rabbit IgG and rabbit anti-mouse IgG) were purchased from Zhong Shan Jin Qiao Co. (Beijing, P.R. China).

### ShRNA Construction

The adenoviral vector AdshRNA was utilized to construct interference plasmids targeting SKAP2 (AdshRNA–SKAP2) and cortactin (AdshRNA–cortactin). Adenovirus encapsulation was performed using helper plasmids psPAX2 (Addgene #12260) and pMD2.G (Addgene #12259). The packaged adenovirus was then used to infect cells. An appropriate amount of polybrene (final concentration of 10ug/mL) was added to increase the efficiency of virus infection of cells. Ovarian tissues were cultured in α-MEM medium supplemented with 3 mM glutamine, 0.1% bovine serum albumin (BSA), and 1% streptomycin-penicillin. After 96 h of viral infection, primary oocytes (immature oocytes arrested at GV stage) were isolated, collected, and transferred to M2 medium droplets under mineral oil. The cultures were transferred into an incubator for maintenance (37 °C, 5% CO_2_, 95% humidity) [22, 23]. Sequences of the shRNA constructs are shown in the following table (Table 1).

**Table 1:** Sequences of the shRNA constructs

SCR shRNA	GCCTCGATGATTTCTTCAAGG
SKAP2-493	GCAATAGATGGCTATGATGTC
SKAP2-629	GGGTCCAGCAGCTGAAATTTA
SKAP2-1027	GCCATTGGCTTGGTGCCTAAA
cortactin-80	GGTGCCATCTGCCTATCAGAA
cortactin-307	GAGTACCAGTCGAAGCTTTCC
cortactin-462	GGCCTGACCTATACATCAGAG

### Confocal Microscopy

After 16 h of culture, oocytes were co-stained with antibodies against SKAP2 and WAVE2, and then nuclear staining with 4',6-diamidino-2-phenylindole (DAPI). Oocytes progressing through specific meiotic phases, including metaphase I (MI), anaphase I (ATI), and metaphase II (MII), were identified and subsequently subjected to microscopic examination and image acquisition. Similarly, after 16 h of culture, oocytes were co-stained with antibodies against SKAP2 and cortactin, along with DAPI nuclear staining. MI, ATI, and MII cells were again selected for observation and imaging. Oocytes subjected to SKAP2 knockdown and control groups were co-stained with either WAVE2/F-actin or cortactin/F-actin, then processed and imaged following the above steps [24, 25].

### Light Microscopy

Each cell group's morphology was examined using a light microscope and captured on camera after 16 h of incubation. The proportions of cells exhibiting enlarged polar bodies, symmetric division, and asymmetric division were quantified [26].

### Quantitative Real-Time PCR Analysis

Quantitative real-time PCR (qPCR) combined with the Delta-delta CT (DDCT) method was employed to analyze the expression levels of SKAP2, Cyclin A, Cyclin B, CDK1, and PLK1 genes. Total RNA was extracted from 50 oocytes using the Dynabead mRNA Direct Kit (Life Technologies Ltd., Oslo, Norway). First-strand cDNA was synthesized using Oligo (dT) 12–18 primers (Takara Bio Inc., Tokyo, Japan) and a cDNA Synthesis Kit (Toyobo, Osaka, Japan). The cDNA fragments were then amplified using gene-specific primers, as listed below (Table 2). Quantitative real-time PCR (qPCR) was performed using a StepOne Real-Time PCR System (Applied Biosystems, Foster City, CA, USA) with SYBR Green Realtime PCR Master Mix (Life Technologies, Carlsbad, CA, USA). The thermal cycling settings were arranged as follows: an initial two—stage denaturation, first at 50 °C for 2 min and then at 95 °C for 2 min. Subsequently, 40 cycles were carried out, each involving a 15—second denaturation at 95 °C and a 1—minute combined annealing and extension at 60 °C [27, 28]. Primer specificity was validated using the NCBI Primer-BLAST tool to ensure target specificity and prevent nonspecific amplification.

**Table 2:** Gene-specific primers were used to amplify cDNA fragments

Name	Sequence(5’−3’)	Size
GAPDH	AAGAGGGATGCTGCCCTTAC	119 bp
	TACGGCCAAATCCGTTCACA	
SKAP2	TCTGCAAGTGCCTTGGAACA	142 bp
	CCCATCACTGCCTGCTCAAT	
Cyclin A	GTCCTAACGCTCCCATCTCC	72 bp
	TCGGAAAGAGTGTCAGCCTC	
Cyclin B	AGGCCGTGACAAAGGCATAA	180 bp
	CCGTTAGCCTAAACTCAGAAGC	
CDK1	AAGTGTGGCCAGAAGTCGAG	97 bp
	TCGTCCAGGTTCTTGACGTG	
PLK1	TTGAAGGGGTTGCTGTGTGT	91 bp
	TTCATACAGTGGGCTGAGCG	

### Immunofluorescence and Western Blotting (WB) Band Intensity Analysis

Fluorescence intensity was quantified using ImageJ software. To ensure consistent imaging conditions, experimental and control oocyte groups were co-mounted on the same slides. After immunostaining, specific regions of interest (ROIs) within the captured images were analyzed to perform quantitative fluorescence measurements. Uniform ROI dimensions were systematically applied to separately evaluate membrane-associated and cytoplasmic fluorescence signals. Samples displaying extreme fluorescence values (either excessively high or low) were excluded from the analysis to maintain data integrity. The average fluorescence intensity of treated and control oocytes was compared based on the mean values of all measurements [29]. For immunoblotting data quantification, band intensities were measured using ImageJ. All analyses were performed in triplicate.

### Immunohistochemistry (IHC)

Ovarian tissues from 4–6-week-old ICR female mice were collected to evaluate SKAP2 expression. Paraffin-embedded tissue sections underwent sequential processing for deparaffinization and rehydration as follows: three 15-min xylene baths (I, II, III); two 5-min anhydrous ethanol incubations (I, II); 5-min immersions in 85% and 75% ethanol gradients; followed by distilled water rinsing to complete tissue rehydration. For antigen retrieval, samples were microwaved in citrate buffer (PH6.0) at medium heat for 8 min, followed by an 8-min ceasefire, 7 min at low heat, and natural cooling. Subsequently, the samples were rinsed three times with phosphate-buffered saline (PBS) (PH7.4) for 5 min each. To block endogenous peroxidase activity, tissue sections were incubated in 3% hydrogen peroxide for 25 min and rinsed three times with PBS (PH7.4) for 5 min each. For serum blocking, tissues were incubated at room temperature with 3% BSA for 30 min. Primary antibody solutions were prepared in PBS (PH7.4) and carefully administered to tissue sections using dropwise application, followed by 12–16 h incubation at 4 °C. After extensive PBS (PH7.4) washing, HRP-conjugated secondary antibodies were applied using the same dropwise technique and maintained at ambient temperature for 50 min. Hematoxylin was used to restain the nuclei of the cells. Tissue sections underwent sequential dehydration and clearing through graded ethanol solutions followed by xylene treatment. The protocol consisted of: 5-min immersions in 75% and 85% ethanol; two 5-min incubations in anhydrous ethanol (I, II); and 5-min xylene I baths. After brief air-drying, sections were permanently mounted with neutral balsam to complete histological processing [30].

### Data Analysis

Quantitative data analysis was conducted utilizing GraphPad Prism (GraphPad Software, Inc., California, USA). Statistical significance between experimental groups was assessed using Student's t-test and one-way analysis of variance (ANOVA), with a predetermined threshold of *p* < 0.05 indicating statistical significance [31].

## Results

### Ovarian Tissue SKAP2 Expression and Localization

**Fig. 1:**
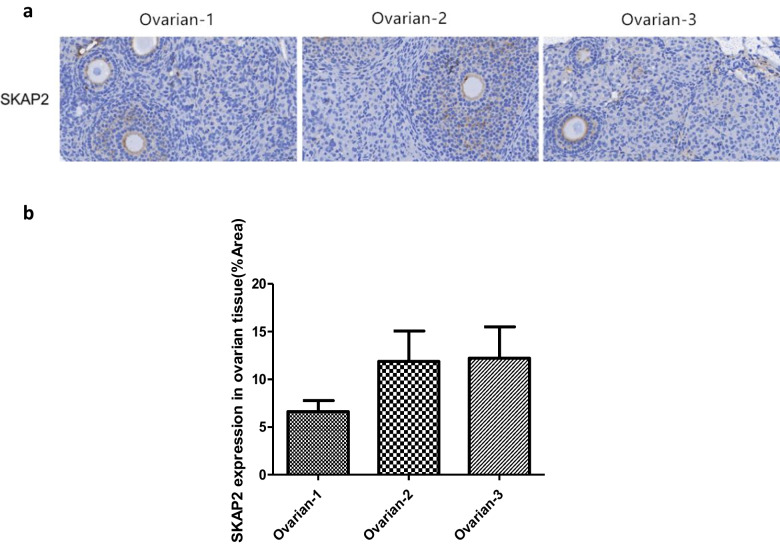
SKAP2 expression in ovarian tissues was detected by immunohistochemistry. The scale bar of Fig. 1**a** shows 40 μm. Immunohistochemical analysis in Fig. 1a demonstrates nuclear staining with hematoxylin (blue) and positive protein expression visualized by DAB chromogen (brown). As shown in Fig. 1**b**, SKAP2 was stably expressed in mouse ovarian tissues, and its expression was markedly up-regulated especially in the follicular region rich in oocytes. All experiments were repeated at least three times. Data are shown as the mean ± standard deviation

**Fig. 2:**
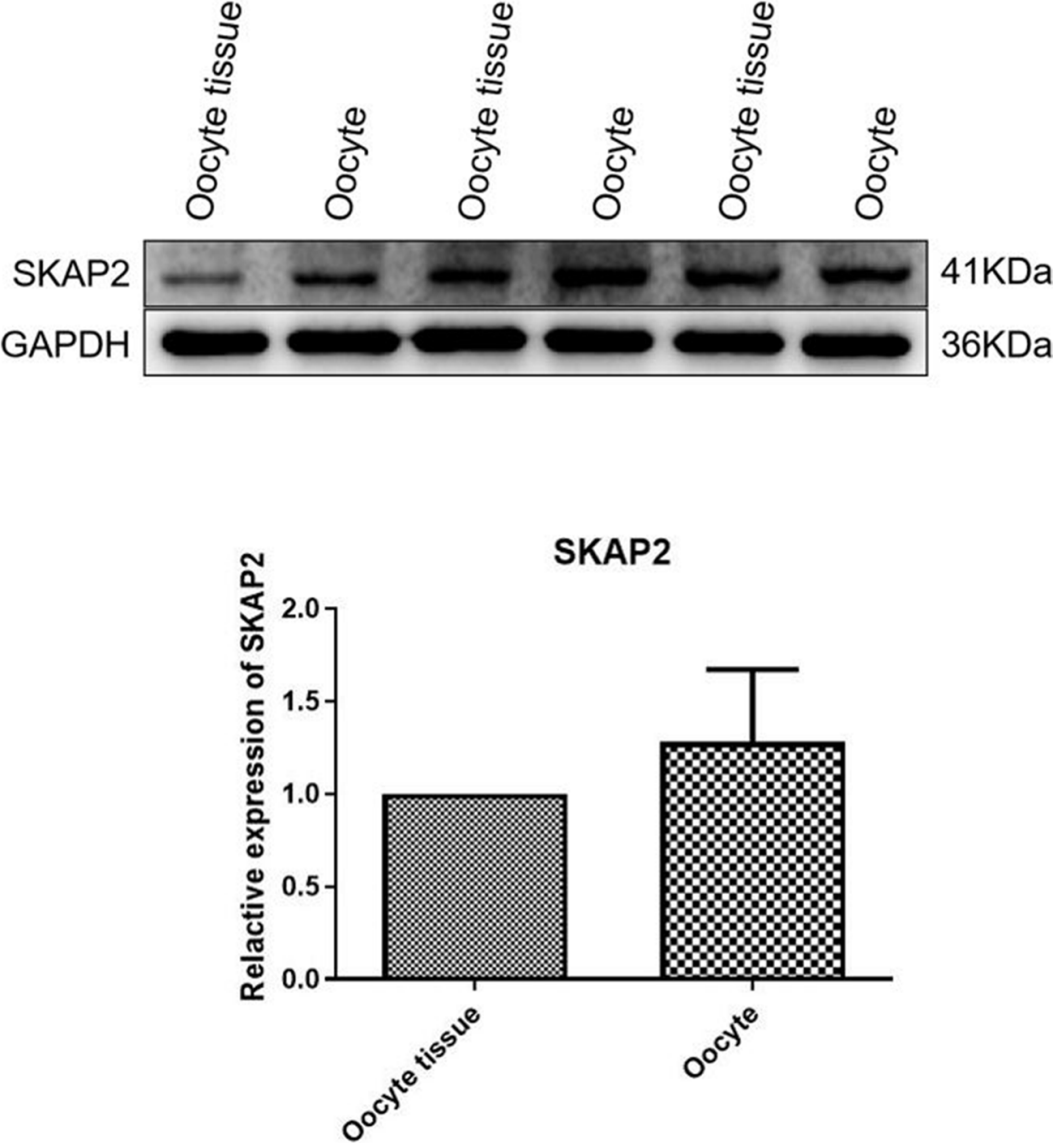
SKAP2 expression in ovarian tissue and oocytes was detected by Western Blot. As shown in Fig. 2, SKAP2 was stably expressed in both mouse ovarian tissues and oocytes, and both SKAP2 proteins showed higher abundance expression levels in oocytes compared to ovarian tissues. All experiments were repeated at least three times. Data are shown as the mean ± standard deviation

Ovarian tissues from 4–6-week-old ICR female mice were collected to examine SKAP2 expression and its distribution in oocytes. SKAP2 exhibited stable expression in mouse ovarian tissues, as shown in Fig. 1. WB analysis was performed to detect SKAP2 expression in both ovarian tissues and oocytes. Consistent with the tissue findings, SKAP2 was also stably expressed in mouse oocytes, as demonstrated in Fig. 2.

### Impact of SKAP2 and Cortactin Depletion on Meiotic Progression and Associated Molecular Pathways in Oocytes

**Fig. 3:**
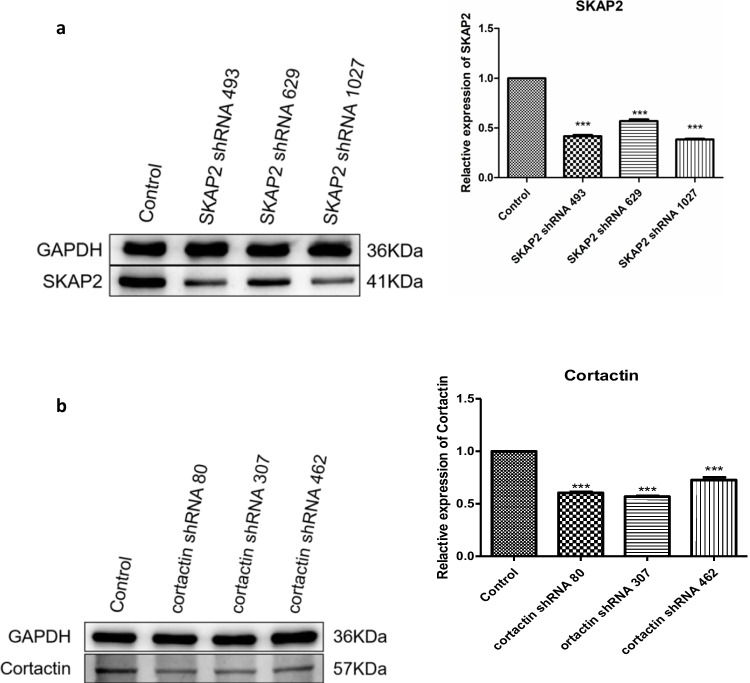
Western blot analysis was employed to evaluate the knockdown efficiency of adenoviral vectors encoding three distinct shRNA sequences targeting SKAP2 (shRNA-493, 629, 1027) and cortactin (shRNA-80, 307, 462). Figure 3**a** demonstrates a substantial reduction in SKAP2 protein levels following transduction with SKAP2-specific shRNA constructs, particularly with shRNA-1027 achieving the most effective suppression. Parallel experiments with cortactin-targeting shRNAs revealed pronounced decreases in protein expression, where shRNA-307 showed the highest silencing efficacy, as documented in Fig. 3**b**. All experiments were repeated at least three times. Data are shown as the mean ± standard deviation. One-way analysis of variance (ANOVA) was used for data analysis. Experimental data were normalized to the control group mean (set to 1.0). All experimental replicates were expressed relative to the control group mean rather than absolute values. *P < 0.05, ** *P* < 0.01, *** *P* < 0.001, and **** *P* < 0.0001

**Fig. 4:**
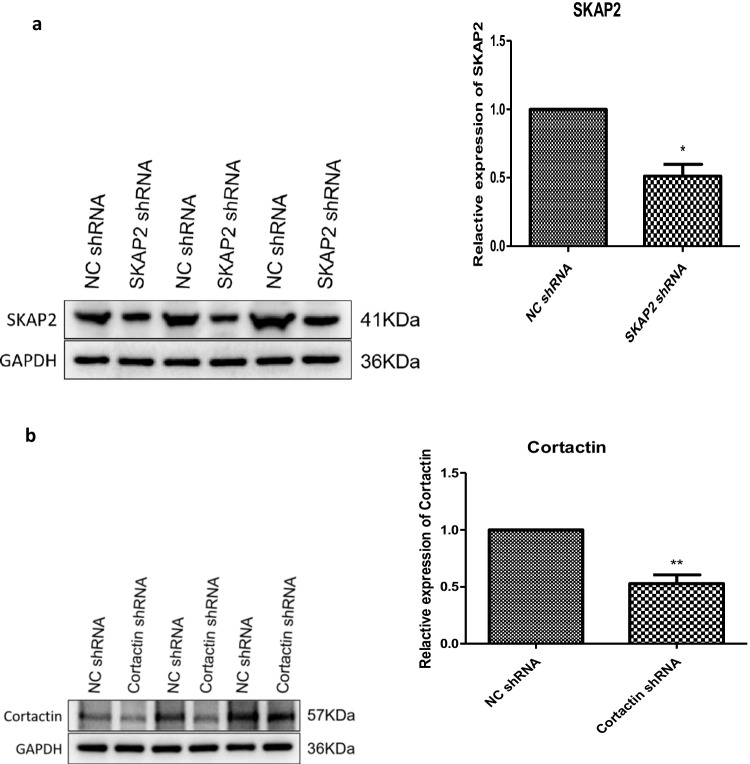
To validate the knockdown efficiency of shRNA-SKAP2 (shRNA-1027) and shRNA-cortactin (shRNA-307) adenoviruses in mouse ovarian tissues, three experimental groups were infected with the respective adenoviral constructs, followed by oocyte isolation. As shown in Fig. 4**a**, Western blot analysis revealed significant reductions in SKAP2 expression in oocytes derived from shRNA-SKAP2-treated ovarian tissues across all groups. Similarly, as shown in Fig. 4**b**, cortactin expression was markedly decreased in oocytes from shRNA-cortactin-treated tissues. All experiments were repeated at least three times. Data are shown as the mean ± standard deviation. The Student’s t-test was used for data analysis. Experimental data were normalized to the control group mean (set to 1.0). All experimental replicates were expressed relative to the control group mean rather than absolute values. *P < 0.05, ** *P* < 0.01, *** *P* < 0.001, and **** *P* < 0.0001

**Fig. 5:**
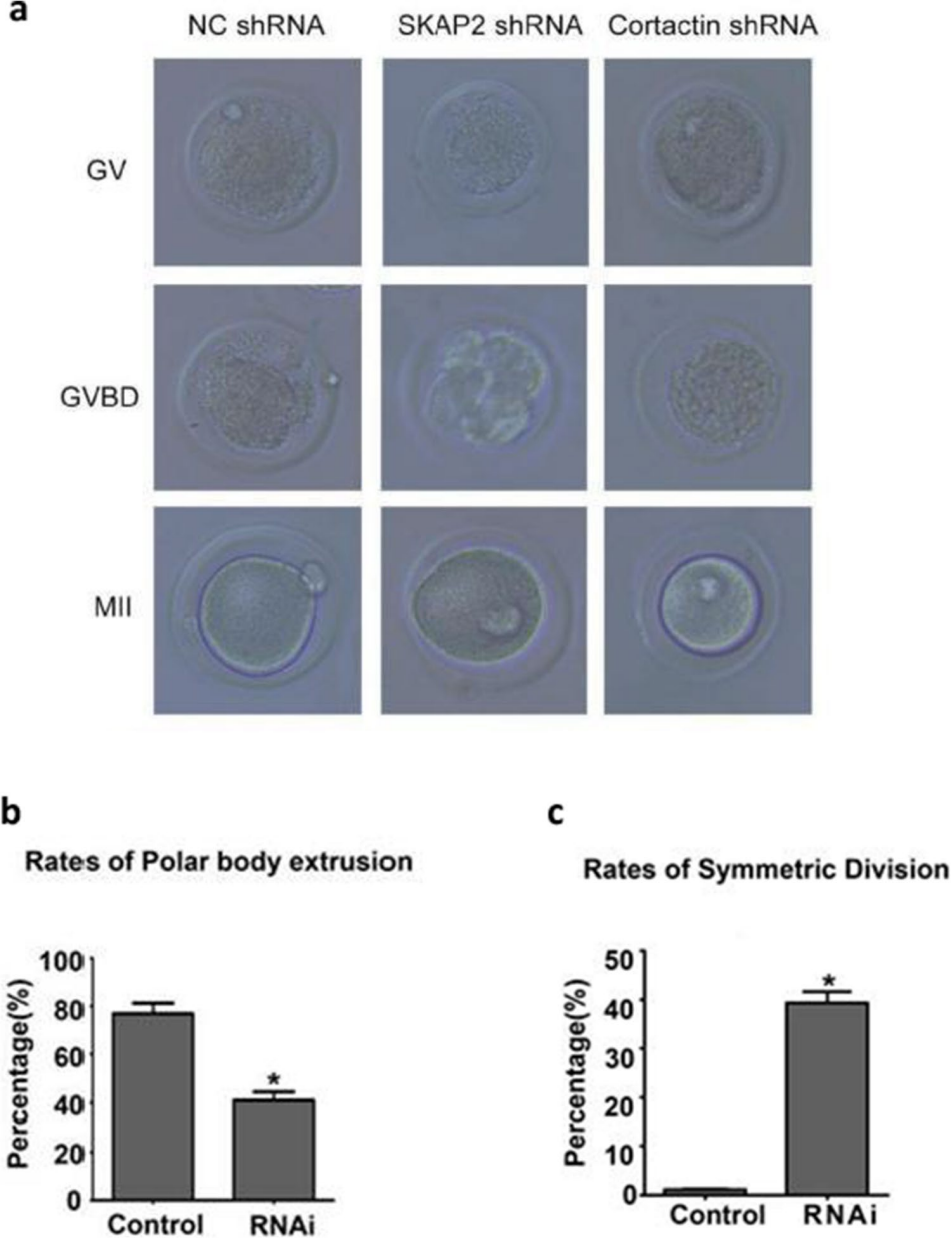
As illustrated in Fig. 5**a**, Knockdown of SKAP2 and cortactin protein expression levels by SKAP2 shRNA adenovirus and cortactin shRNA adenovirus resulted in a lower capacity for cellular first polar body extrusion than controls. Quantitative analysis revealed that SKAP2 knockdown significantly decreased first polar body extrusion compared to control groups (Fig. 5**b**). Furthermore, oocytes subjected to SKAP2 silencing exhibited a marked increase in symmetric division frequency relative to controls (Fig. 5**c**). All experiments were repeated at least three times. Data are shown as the mean ± standard deviation. The Student’s t-test was used for data analysis. *P < 0.05, ** *P* < 0.01, *** *P* < 0.001, and **** *P* < 0.0001

WB analysis was conducted to assess the efficacy of lentivirus-mediated shRNA interference targeting SKAP2 and cortactin. SKAP2 expression was significantly reduced following shRNA–SKAP2 lentivirus treatment, and cortactin expression was similarly decreased after shRNA–cortactin lentivirus treatment (Fig. 3). These changes were statistically significant. WB analysis further confirmed the effectiveness of shRNA–SKAP2 and shRNA–cortactin lentivirus interference, with significant reductions in SKAP2 and cortactin expression observed (Fig. 4). These results were also statistically significant. Oocytes were cultured to the GV, GVBD, and MII stages. The morphology of each cell group was examined using light microscopy. Compared to the control group, oocytes treated with SKAP2 shRNA or cortactin shRNA lentivirus exhibited impaired polar body extrusion (Fig. 5).

### Cyclin Expression Assay

**Fig. 6:**
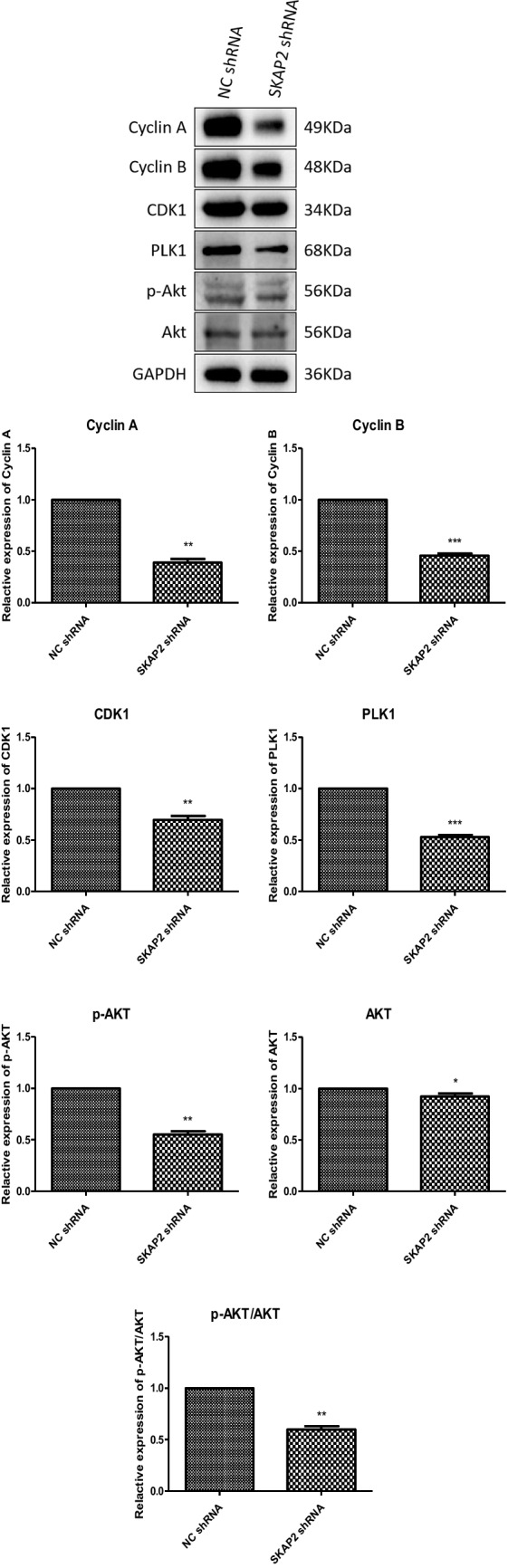
Western blot analysis of protein levels of cell cycle protein A, cell cycle protein B, CDK1, PLK1, Akt and phosphorylated Akt (p-Akt) was performed in oocytes. As shown in Fig. 6, the expression of key cell cycle regulators (cyclin A, cyclin B, CDK1, PLK1), as well as Akt and p-Akt, was significantly reduced in SKAP2 knockout oocytes compared with control oocytes. All experiments were repeated at least three times. Data are shown as the mean ± standard deviation. The Student’s t-test was used for data analysis. Experimental data were normalized to the control group mean (set to 1.0). All experimental replicates were expressed relative to the control group mean rather than absolute values. *P < 0.05, ** *P* < 0.01, *** *P* < 0.001, and **** *P* < 0.0001

**Fig. 7:**
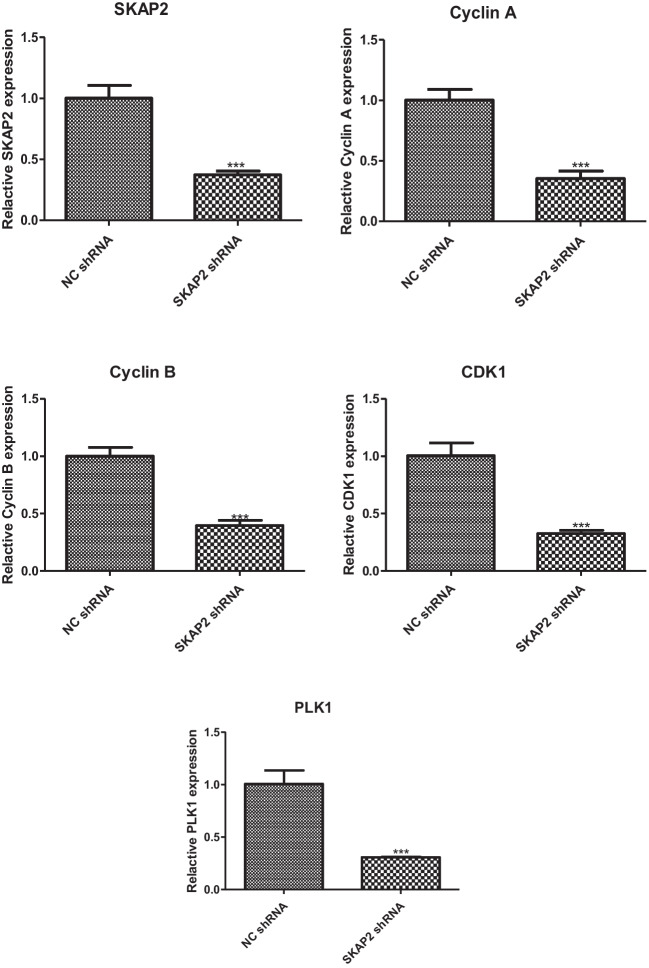
Quantitative real-time PCR (qRT-PCR) was employed to assess the mRNA levels of SKAP2 and cell cycle regulators (Cyclin A, Cyclin B, CDK1, PLK1) in oocytes. As demonstrated in Fig. 7, SKAP2 knockdown oocytes exhibited significantly reduced mRNA expression of SKAP2 and these regulators compared to control groups. All experiments were repeated at least three times. Data are shown as the mean ± standard deviation. The Student’s t-test was used for data analysis. *P < 0.05, ** *P* < 0.01, *** *P* < 0.001, and **** *P* < 0.0001

The protein expression levels of Cyclin A, Cyclin B, CDK1, PLK1, phosphorylated Akt (p-Akt), and total Akt were analyzed by Western blot. Relative to control samples, experimental groups subjected to SKAP2 shRNA treatment demonstrated markedly decreased protein levels of key cell cycle regulators (Cyclin A, Cyclin B, CDK1, PLK1) Akt and phosphorylated Akt, as shown in Fig. 6. These differences were statistically significant. While Western blot analyses revealed minimal visual differences in Akt, p-Akt and CDK expression patterns, Quantitative validation of GAPDH expression (used as a loading control) confirmed its stability across all experimental groups. Grayscale densitometric analysis demonstrated statistically significant reductions in Akt, p-Akt, and CDK expression levels compared to controls (Student’s t-test: P = 0.0449, P = 0.0019, and P = 0.0055, respectively). The mRNA levels of SKAP2, Cyclin A, Cyclin B, CDK1, and PLK1 were quantified by the reverse transcription-quantitative polymerase chain reaction (RT-qPCR). Relative to control samples, the SKAP2 shRNA group showed decreased mRNA levels of SKAP2, Cyclin A, Cyclin B, CDK1, and PLK1 (Fig. 7). These results were also statistically significant.

### Immunofluorescence Detection of SKAP2–WAVE2, Cortactin Binding Expression

**Fig. 8:**
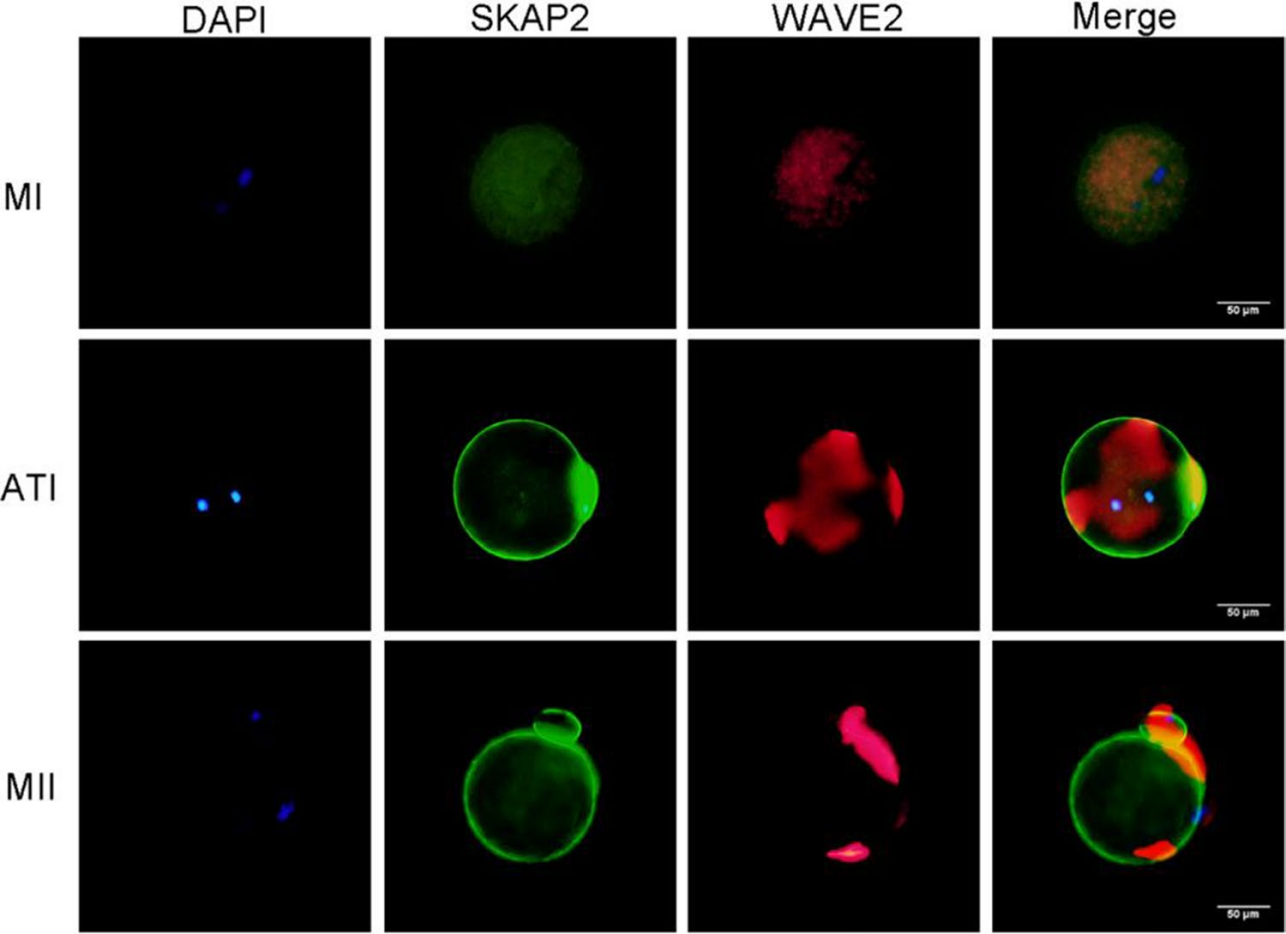
Immunofluorescence analysis was performed to assess the spatial co-localization of SKAP2 and WAVE2 during oocyte meiotic maturation. As shown in Fig. 8, SKAP2 exhibited predominant localization in the proximal cortical region of oocytes from metaphase I (MI) to metaphase II (MII) stages. Furthermore, SKAP2-WAVE2 co-localization displayed stage-specific patterns: (1) at the MI stage, concentrated asymmetrically at the cell membrane periphery; (2) during anaphase I (AI), distributed along the emergent cell membrane; and (3) at the MII stage, localized specifically at the intercellular junction between daughter cells. All experiments were repeated at least three times

**Fig. 9:**
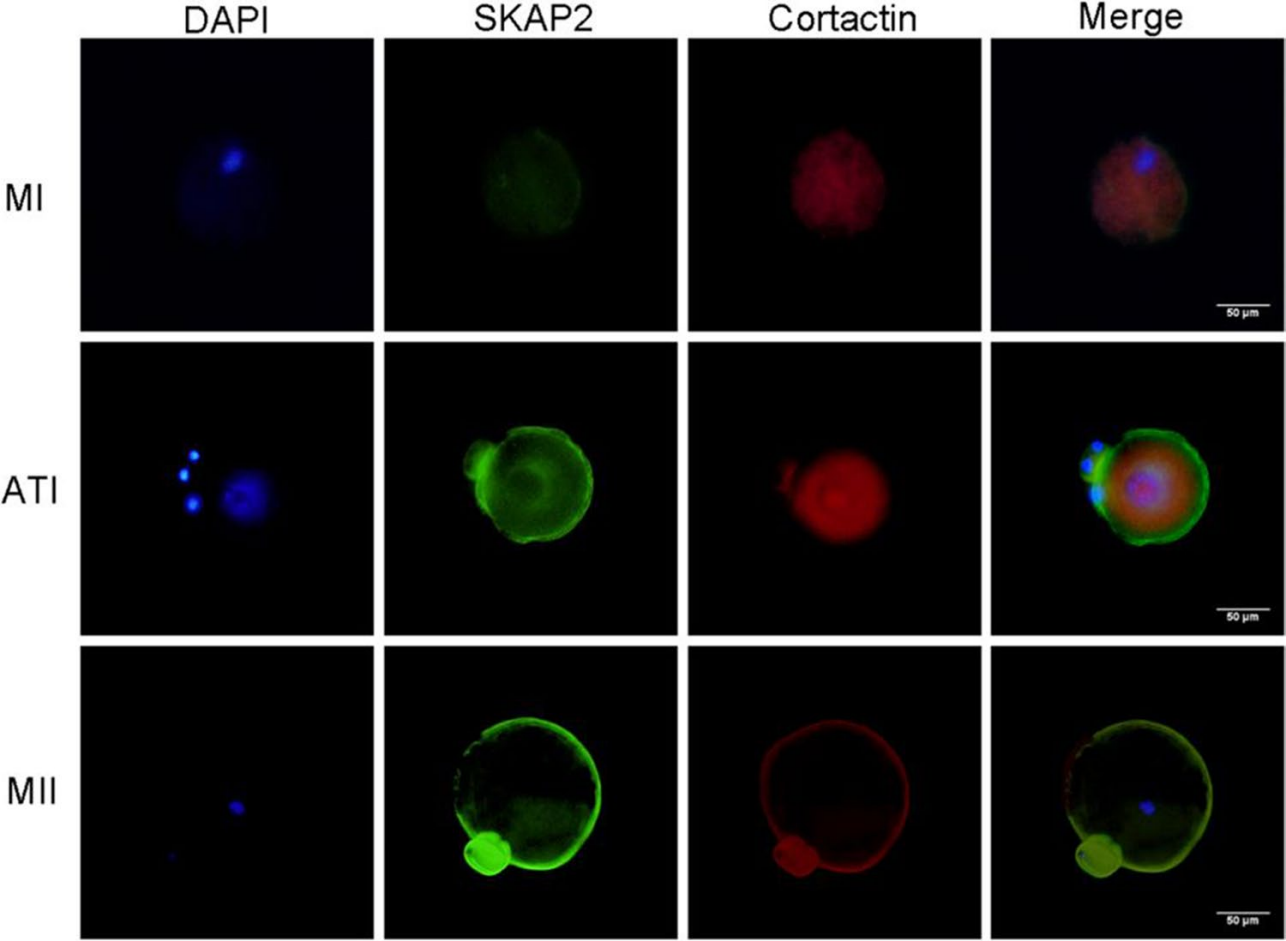
Immunofluorescence analysis was performed to assess the spatial co-localization of SKAP2 and cortactin during oocyte meiotic maturation. As shown in Fig. 9, SKAP2-cortactin co-localization displayed stage-specific patterns: (1) at the MI stage, concentrated asymmetrically at the cell membrane periphery; (2) during anaphase I (AI), distributed along the emergent cell membrane; and (3) at the MII stage, localized specifically at the intercellular junction between daughter cells. All experiments were repeated at least three times

**Fig. 10:**
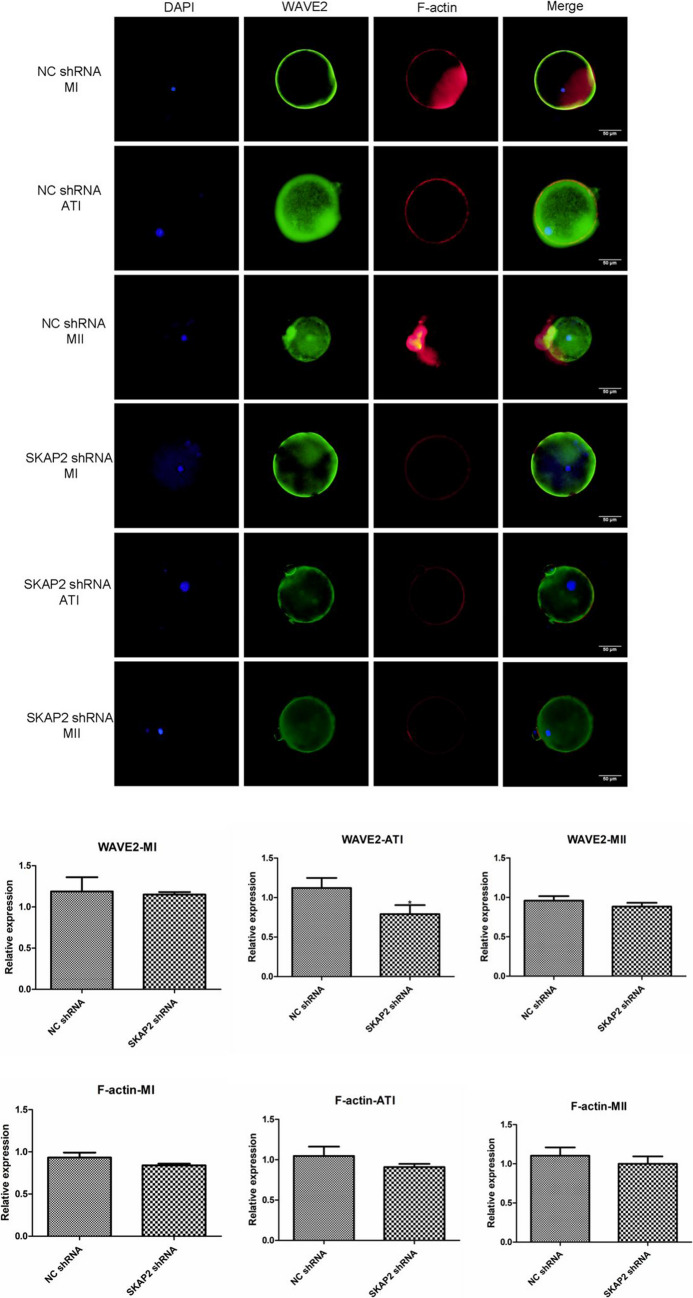
To investigate SKAP2-mediated regulation of WAVE2 membrane dynamics, immunofluorescence analysis was performed in control and SKAP2 knockdown oocytes. As shown in Fig. 10, in control groups, WAVE2 exhibited stable expression and cortical localization throughout meiotic progression, with stage-specific co-localization patterns with F-actin: (1) asymmetric cortical clustering at metaphase I (MI), (2) peripheral membrane distribution during anaphase I (AI), and (3) intercellular junction accumulation at metaphase II (MII). However, SKAP2 depletion significantly reduced WAVE2 expression levels and disrupted its co-localization with F-actin across all meiotic stages. All experiments were repeated at least three times. Data are shown as the mean ± standard deviation. The Student’s t-test was used for data analysis. *P < 0.05, ** *P* < 0.01, *** *P* < 0.001, and **** *P* < 0.0001

**Fig. 11:**
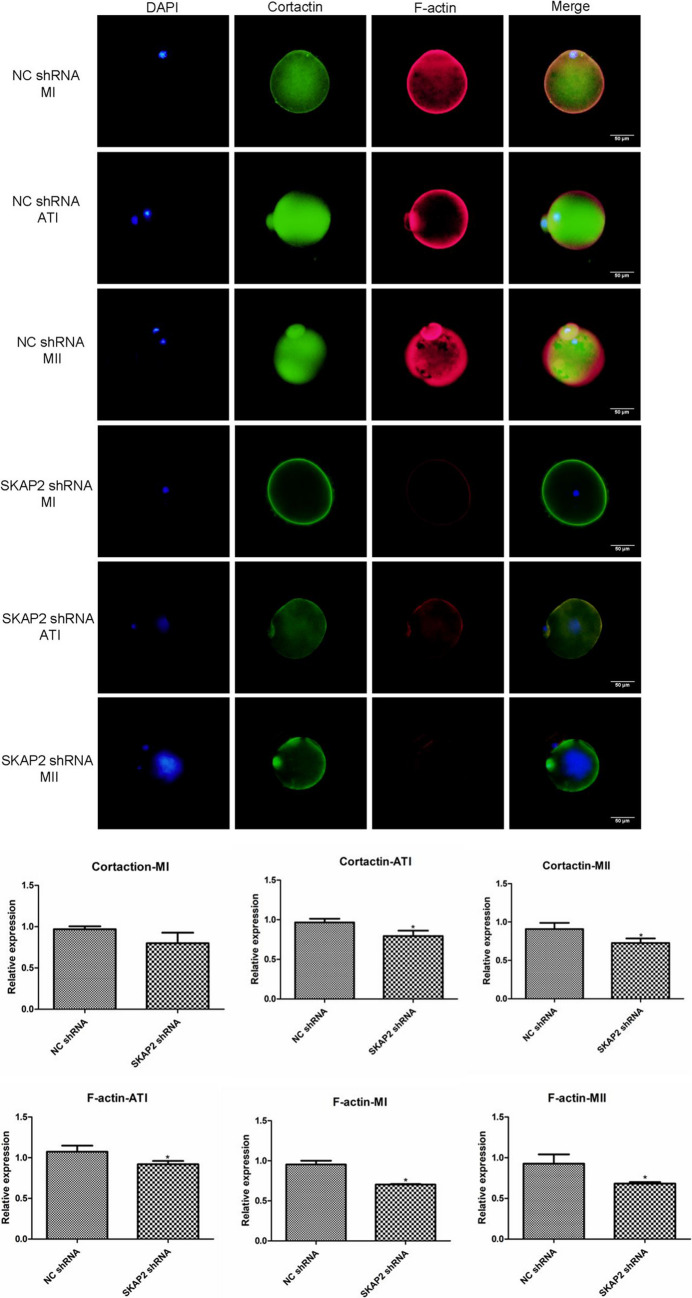
To investigate SKAP2-mediated regulation of cortactin membrane dynamics, immunofluorescence analysis was performed in control and SKAP2 knockdown oocytes. As shown in Fig. 11, in control groups, cortactin exhibited stable expression and cortical localization throughout meiotic progression, with stage-specific co-localization patterns with F-actin: (1) asymmetric cortical clustering at metaphase I (MI), (2) peripheral membrane distribution during anaphase I (AI), and (3) intercellular junction accumulation at metaphase II (MII). However, SKAP2 depletion significantly reduced cortactin expression levels and disrupted its co-localization with F-actin across all meiotic stages. All experiments were repeated at least three times. Data are shown as the mean ± standard deviation. The Student’s t-test was used for data analysis. *P < 0.05, ** *P* < 0.01, *** *P* < 0.001, and **** *P* < 0.0001

The results revealed the subcellular localization of SKAP2 during meiosis. From the metaphase I (MI) to metaphase II (MII) stages, SKAP2 was predominantly localized in the cortical region and proximal cortex of oocytes (Figs. 8 and 9). To investigate SKAP2's influence on WAVE2 and cortactin localization patterns, immunofluorescence staining was conducted to visualize their membrane-associated distributions. Both WAVE2 and cortactin exhibited stable expression and colocalization with microfilaments in mouse oocyte meiosis. Following SKAP2 downregulation, the average fluorescence intensity of the cell membrane and cytoplasmic WAVE2 and cortactin significantly decreased compared to the control group (Figs. 10 and 11).

## Discussion

This study systematically investigated the transcriptional profile and subcellular distribution of SKAP2 during meiotic progression. SKAP2 protein levels in both ovarian tissues and oocytes from 4–6-week-old ICR strain female mice were quantitatively assessed through IHC and WB. The experimental procedures included paraffin sectioning, antigen retrieval, peroxidase blocking, serum blocking, primary and secondary antibody incubation, DAB staining, hematoxylin counterstaining, and dehydration. The experimental groups comprised a control lentivirus group, an SKAP2 shRNA lentivirus group, and a cortactin shRNA lentivirus group. The expression and distribution of cyclins, SKAP2, WAVE2, and cortactin, as well as the observation of cell morphology, revealed that SKAP2 plays a critical role in oocyte maturation.

According to recent research, patients who have an missed abortion had lower levels of SKAP2 and WAVE2 expression in their chorionic villi than those who undergo an active abortion. This implies that SKAP2 may influence trophoblast cell migration and proliferation, perhaps contributing to missed abortion [32]. Another study showed that reducing SKAP2 activity had distinct impacts on cytoskeletal organization, meiotic spindle translocation, polar body formation, asymmetric cytoplasmic partitioning, and the regulation of ARP2/WAVE2 complex expression. The interaction of SKAP2 with WAVE2 is crucial for the processes of actin assembly and spindle migration, and the SKAP2-mediated Arp2/3 complex activation is indispensable for establishing cytoplasmic asymmetry that occurs during the maturation of mouse oocytes [15].

The adaptor molecule SKAP2 demonstrates ubiquitous expression across various cellular populations and plays crucial roles in numerous intracellular signal transduction cascades. According to recent research, SKAP2 plays a critical role in immune responses, cell motility, and cytoskeletal remodeling. Its stable expression and localization in ovarian tissues suggest its importance in oocyte maturation and development [33, 34].

Both SKAP2 and cortactin are cytoskeleton-associated proteins. SKAP2 regulates cell motility through signaling pathways, while cortactin directly modulates microfilament dynamics. Knockdown of SKAP2 and cortactin impaired polar body extrusion in oocytes, indicating their essential roles in oocyte maturation and division. Previous studies suggest that these proteins regulate microfilament stability and dynamics, thereby influencing polar body extrusion [35, 36].

Polar body extrusion abnormalities during oocyte maturation manifest broad biological impacts, critically influencing genomic integrity, embryogenesis, and fertility potential. As an essential meiotic checkpoint governing chromatid separation, defective polar body discharge leads to aberrant retention of redundant chromosomes. This genomic instability predisposes zygotes to mitotic segregation errors, elevating risks of embryonic lethality, congenital malformations, and heritable chromosomal disorders [37]. The extrusion process serves as a biochemical hallmark of meiotic competency. Its impairment frequently causes meiotic arrest at prophase I, diminishing oocyte fertilization capacity and clinical pregnancy rates. Persistent extrusion deficiencies further correlate with compromised oocyte integrity, contributing to recurrent implantation failure, idiopathic infertility, and suboptimal performance in assisted reproduction procedures [38, 39]. These observations highlight the fundamental necessity to decipher SKAP2-dependent regulatory networks in gamete maturation. Mechanistic insights into this pathway may inform innovative ART optimization strategies and prophylactic interventions against transgenerational genetic diseases.

Cell cycle proteins, including cyclin A, cyclin B, CDK1, and PLK1, are essential for cell cycle regulation. The knockdown of SKAP2 greatly lowered the expression of these proteins, suggesting that SKAP2 regulates oocyte maturation by modulating cell cycle protein expression. Additionally, reduced level of p-Akt indicate SKAP2's involvement in the PI3K/Akt signaling pathway, which affects cell survival and cycle progression [40, 41].

Immunofluorescence assays revealed that SKAP2 colocalizes with WAVE2 and cortactin in the proximal cortex and cortical region during oocyte meiosis. SKAP2 depletion markedly reduced cortactin/WAVE2 expression and disrupted their colocalization with F-actin, resulting in diffuse cytoplasmic distribution without membrane specificity. These findings collectively suggest that SKAP2 regulates cytoskeletal dynamics through cortactin/WAVE2 interactions to mediate oocyte maturation [42, 43]. Existing literature indicates that genetic ablation of WAVE2 compromises meiotic chromosome congression, spindle translocation, and polar body emission. Mechanistically, WAVE2 deficiency disrupts γ-tubulin recruitment and MAPK signaling—critical mediators of microtubule organization—culminating in aberrant spindle organization and failed cytoplasmic extrusion during oocyte maturation. These collective observations imply that SKAP2-WAVE2 functional crosstalk may coordinate spindle architecture regulation and meiotic progression in developing oocytes [44]. Future studies will implement quantitative spatial distribution analyses of membrane-localized versus cytoplasmic pools to further elucidate this mechanism.

Our experiment also has some shortcomings and limitations. The ICR mouse strain (outbred, CD-1) was selected for this investigation based on its established utility in pharmacological and toxicological studies. This model organism demonstrates high genetic heterogeneity, robust environmental adaptability, and simplified husbandry requirements. While alternative strains could enhance experimental generalizability, current resource allocation and institutional ethics review timelines precluded additional model incorporation. Future investigations will employ multi-strain validation protocols to assess target antigen expression conservation.

In summary, SKAP2 is stably expressed in mouse ovarian tissues and oocytes and regulates oocyte maturation by modulating the expression and localization of cell cycle proteins and cytoskeleton-associated proteins, including WAVE2 and cortactin. These findings provide an important basis for further understanding of the function of SKAP2 in oocyte development.

Our results demonstrate that SKAP2 is not only significantly expressed in ovarian tissues and oocytes but that its knockdown profoundly affects the maturation process of oocytes and the expression of cell cycle proteins. During murine oocyte development, SKAP2 plays a critical regulatory role by modulating Arp2/3 complex activity through its interaction with WAVE2, which is essential for establishing cytoplasmic asymmetry and polar body extrusion. Overall, SKAP2 plays a key role in oocyte maturation, affecting oocyte division by regulating cell cycle proteins and interacting with WAVE2 and cortactin.

## Supplementary Information

Below is the link to the electronic supplementary material.

Supplementary file1 (DOCX 192 KB)

Supplementary file2 (XLSX 1009 KB)

Supplementary file3 (DOCX 365 KB)

## Data Availability

The data presented in this study are available upon written request from the corresponding author explaining the use and purposes.
