# Supplementary material for: Expression and Regulatory Roles of SKAP2 and Cortactin in Mouse Ovarian Tissue and Oocyte Maturation
Source: Reprod Sci. 2025 Jul 8;32(8):2763–78. doi: 10.1007/s43032-025-01925-4 (PMC12361339; doi:10.1007/s43032-025-01925-4)
Supplement: Supplementary file 1 — Supplementary file1 (DOCX 192 KB) [file 43032_2025_1925_MOESM1_ESM.docx]

**Microtubule staining**

1. Materials and Methods：Oocytes were separately infected with either SKAP2-targeting shRNA adenovirus or control adenovirus, followed by culture in M2 medium. The subcellular localization of the meiotic spindle was subsequently analyzed via immunofluorescence microscopy.
2. Results


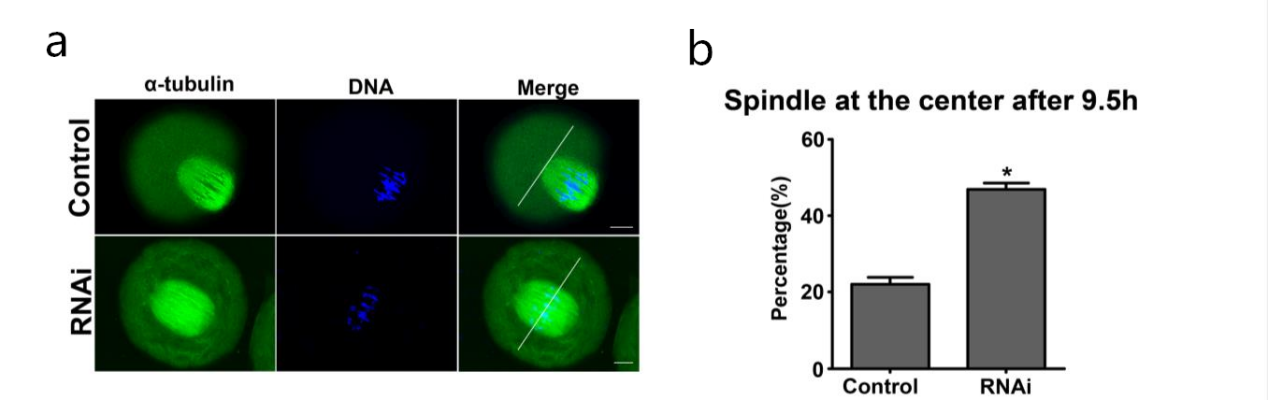


The subcellular localization of meiotic spindles in oocytes is presented in Figure a. In control oocytes, spindle apparatuses predominantly localized peripherally near the cortical region, whereas SKAP2 shRNA-treated oocytes exhibited centralized spindle retention. Immunofluorescence micrographs depict spindle structures (green) and chromosomal DNA morphology (blue), with a scale bar indicating 20 μm. Quantitative analysis in Figure b demonstrates a statistically significant increase in centrally localized spindles in the SKAP2 shRNA group compared to controls ( P < 0.05). All experiments were repeated at least three times. Data are shown as the mean ± standard deviation. The Student’s t-test was used for data analysis.
