# Supplementary figures and images for: Expression and Regulatory Roles of SKAP2 and Cortactin in Mouse Ovarian Tissue and Oocyte Maturation
Source: Reprod Sci. 2025 Jul 8;32(8):2763–78. doi: 10.1007/s43032-025-01925-4 (PMC12361339; doi:10.1007/s43032-025-01925-4)

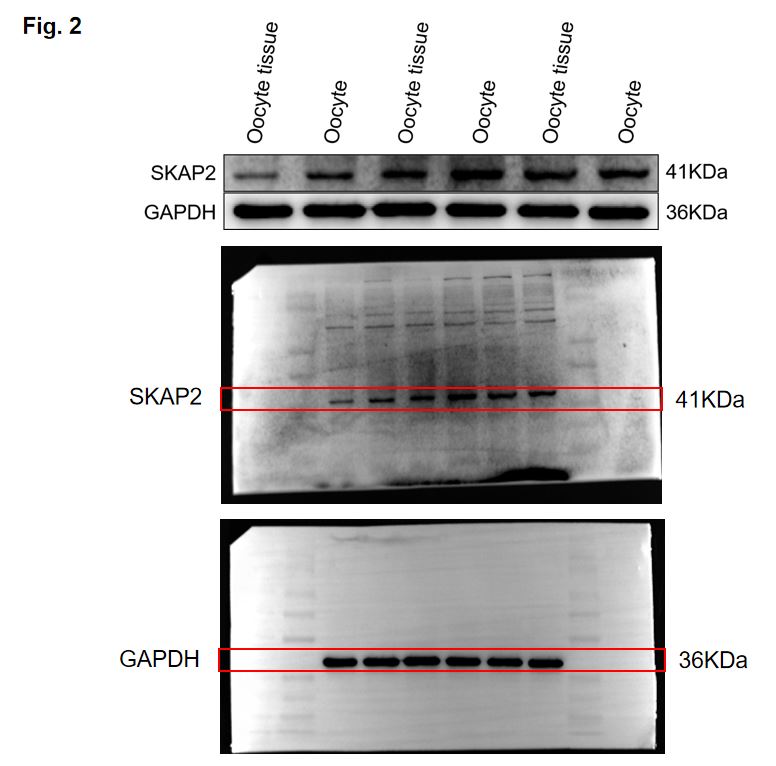


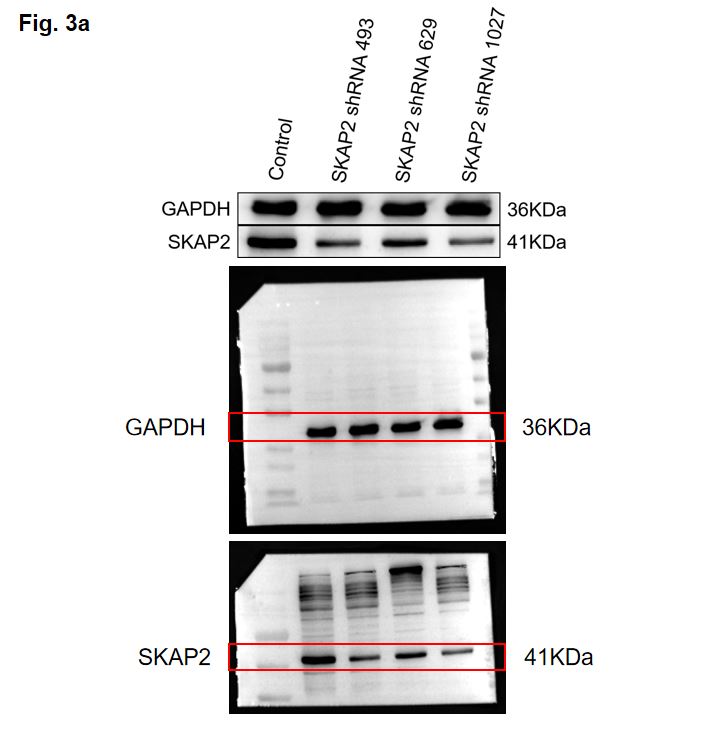


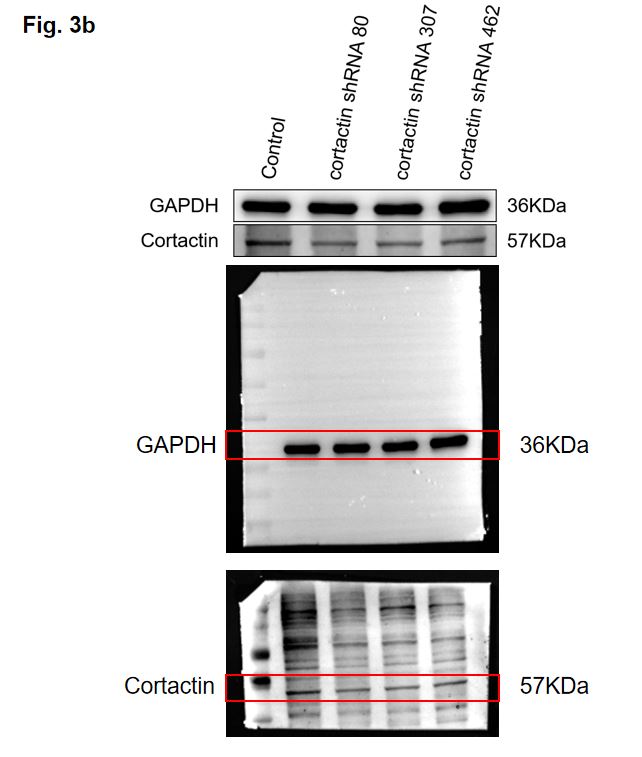


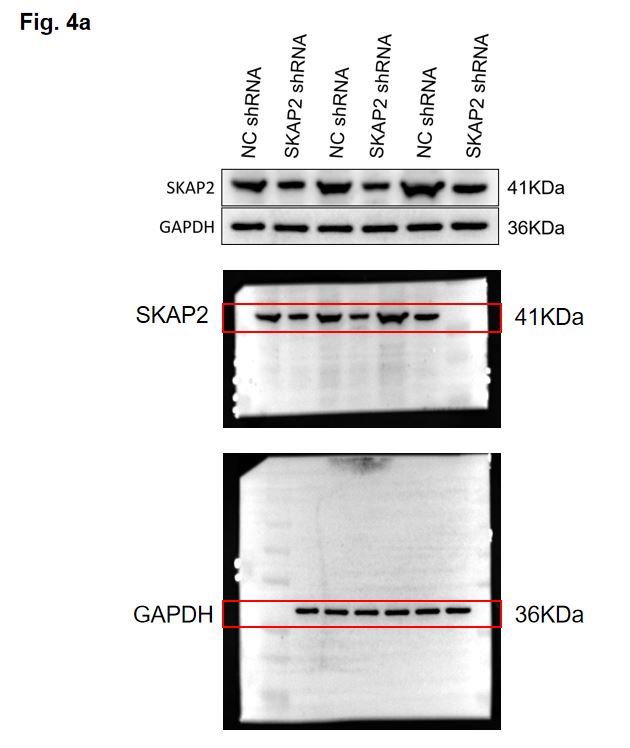


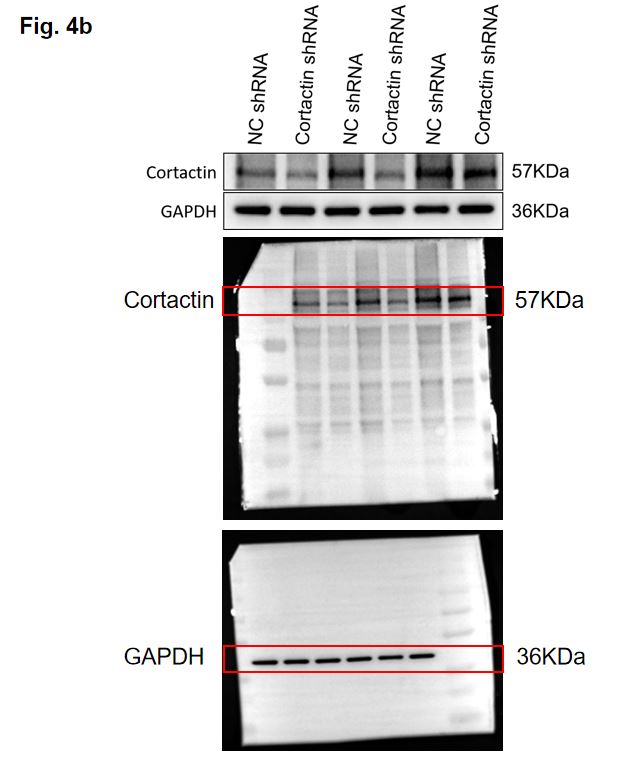


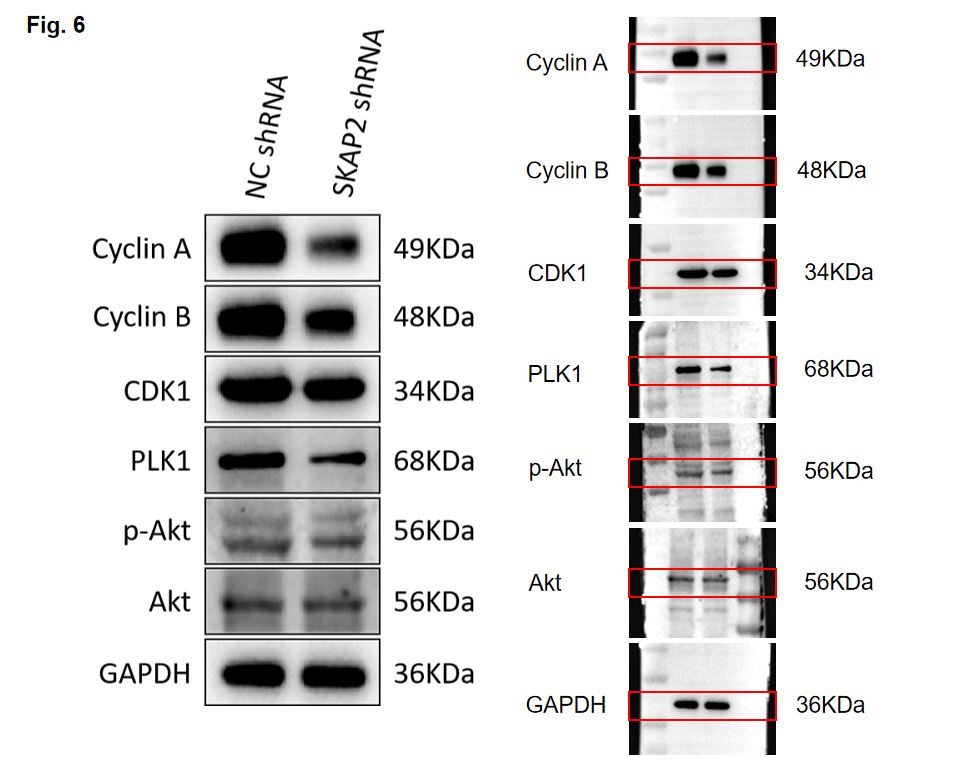

Supplement: Supplementary file 3 — Supplementary file3 (DOCX 365 KB) [file 43032_2025_1925_MOESM3_ESM.docx]
